# Computerized Music-Reading Intervention Improves Resistance to Unisensory Distraction Within a Multisensory Task, in Young and Older Adults

**DOI:** 10.3389/fnhum.2021.742607

**Published:** 2021-09-10

**Authors:** Alexandros T. Karagiorgis, Nikolas Chalas, Maria Karagianni, Georgios Papadelis, Ana B. Vivas, Panagiotis Bamidis, Evangelos Paraskevopoulos

**Affiliations:** ^1^School of Medicine, Faculty of Health Sciences, Aristotle University of Thessaloniki, Thessaloniki, Greece; ^2^School of Music Studies, Faculty of Fine Arts, Aristotle University of Thessaloniki, Thessaloniki, Greece; ^3^Institute for Biomagnetism and Biosignalanalysis, University of Münster, Münster, Germany; ^4^Department of Psychology, CITY College, University of York Europe Campus, Thessaloniki, Greece; ^5^Department of Psychology, University of Cyprus, Nicosia, Cyprus

**Keywords:** music training, EEG, functional connectivity, deviance distraction, aging

## Abstract

Incoming information from multiple sensory channels compete for attention. Processing the relevant ones and ignoring distractors, while at the same time monitoring the environment for potential threats, is crucial for survival, throughout the lifespan. However, sensory and cognitive mechanisms often decline in aging populations, making them more susceptible to distraction. Previous interventions in older adults have successfully improved resistance to distraction, but the inclusion of multisensory integration, with its unique properties in attentional capture, in the training protocol is underexplored. Here, we studied whether, and how, a 4-week intervention, which targets audiovisual integration, affects the ability to deal with task-irrelevant unisensory deviants within a multisensory task. Musically naïve participants engaged in a computerized music reading game and were asked to detect audiovisual incongruences between the pitch of a song’s melody and the position of a disk on the screen, similar to a simplistic music staff. The effects of the intervention were evaluated via behavioral and EEG measurements in young and older adults. Behavioral findings include the absence of age-related differences in distraction and the indirect improvement of performance due to the intervention, seen as an amelioration of response bias. An asymmetry between the effects of auditory and visual deviants was identified and attributed to modality dominance. The electroencephalographic results showed that both groups shared an increase in activation strength after training, when processing auditory deviants, located in the left dorsolateral prefrontal cortex. A functional connectivity analysis revealed that only young adults improved flow of information, in a network comprised of a fronto-parietal subnetwork and a multisensory temporal area. Overall, both behavioral measures and neurophysiological findings suggest that the intervention was indirectly successful, driving a shift in response strategy in the cognitive domain and higher-level or multisensory brain areas, and leaving lower level unisensory processing unaffected.

## Introduction

Our cognitive system is constantly fed with information from multiple sensory channels. In this rich environment, to function effectively, it is important to select goal-relevant information and ignore irrelevant stimuli, while at the same time monitoring the environment for potential threats. Cognitively, this is achieved by the interplay between top-down and bottom-up attentional processes (Itti and Koch, 2001; Corbetta and Shulman, 2002). Bottom-up attentional capture is typically driven by the perceptual saliency of the stimulus (Itti and Koch, 2001), that is sudden, bright, or loud stimuli would have higher weights in the saliency map and win the competition for attentional resources (selection) in a winner-take-all model (Desimone and Duncan, 1995; Desimone, 1998). Top-down control biases selection by modulating perceptual processing (e.g., increasing/decreasing weights) and/or directing attention to relevant locations. At the brain level, distinct frontoparietal networks are responsible for bottom-up and top-down attention (Corbetta and Shulman, 2002). In real-world situations, information must be selected and integrated from different sensory modalities, resulting in a dynamic interplay between attention and multisensory integration (Tellinghuisen and Nowak, 2003; Mozolic et al., 2008; Geitner et al., 2019; Marsja et al., 2019). Recent studies suggest that multisensory integration interacts with both bottom-up and top-down attentional processes. That is, stimuli from different sensory modalities can be automatically integrated as a function of spatial and temporal correspondence, but multisensory integration can also be modulated by top-down attentional selection (Talsma et al., 2010; Talsma, 2015). In other words, both bottom-up, such as the saliency of the sensory stimuli, and top-down factors (e.g., expectations and familiarity) can influence multisensory integration. Relevant to the purpose of this study, the competition of uni-sensory and integrated multisensory stimuli for attentional selection and capture appears to be further influenced by aging (Downing et al., 2015; Broadbent et al., 2018).

Research supports that top-down selective attention is particularly susceptible to age-related decline (Gazzaley et al., 2005). Particularly, older adults are less able, than younger adults, to suppress irrelevant but salient information and consequently are more prone to distraction (Hasher and Zacks, 1988; Gazzaley et al., 2005). However, this higher susceptibility to distraction is not necessarily global, as it has been found to interact with the sensory modality in cross-modal tasks (Guerreiro et al., 2010; Robinson et al., 2018). In particular, it has been suggested that age-related decline is present in the combination of visual distractors during an auditory task but not vice-versa nor within the same modality (Van Gerven and Guerreiro, 2016; but also see Leiva et al., 2015). Nevertheless, studies also support that older adults may able to compensate for cognitive decline by over-recruitment, or over-activation, of frontal areas (Kopp et al., 2014; Swirsky and Spaniol, 2019; Weakley and Schmitter-Edgecombe, 2019; Kardos et al., 2020). However, the effects of age-related cognitive decline and compensatory brain mechanisms on the activity of single frontal brain areas are varied or even contradictory in previous studies (Davis et al., 2008; Park and Reuter-Lorenz, 2009; Nyberg et al., 2010; Morcom and Johnson, 2015; Morcom and Henson, 2018). More consistent results can be seen in functional connectivity studies which have shown age-related impoverishment of in networks involving frontal brain areas and evidence suggests that age-related attentional decline may be attributed to disrupted dorsal attentional networks in older adults (Grady, 2012).

Despite the age-related declines in cognitive and brain function, neuroplasticity seems to be retained in older adults, which suggests that mental training may be effective in producing long-term changes (Bamidis et al., 2014; Bherer, 2015; Chapman et al., 2015). Previous research has shown that training is effective in improving selective attention by reducing attentional capture by task-irrelevant stimuli in older adults, and that these positive effects may be long-lasting (Mozolic et al., 2011; Buitenweg et al., 2012; Mayas et al., 2014; Rienäcker et al., 2018; Wilkinson and Yang, 2020). However, these studies employed training protocols that included various cognitive tasks, either commercial “brain training games” or more established experimental tasks, such as the n-back, or the Go/No-Go, in a uni- or cross-modal sensory context that did not require multisensory integration. Given that multisensory stimuli result in greater attentional capture relative to unisensory stimuli (Downing et al., 2015; Geitner et al., 2019; Marsja et al., 2019), it would be interesting to explore how adding multisensory integration in a training protocol would impact the competition between unisensory and multisensory stimuli in attentional control. However, to our knowledge, this area is largely unexplored, not only in aging, but in younger adults as well.

The present study aims at investigating the effects of a multi-sensory integration training intervention on attentional performance in younger and older adults. Based on the work investigating the neuroplasticity effects of music training by Paraskevopoulos et al. (2012a; 2014; 2015); Pantev et al. (2015), the training intervention employed a computerized music-reading serious game spanning 4 weeks (*MusicPlast*) (Paraskevopoulos et al., 2021). The game was accessible through personal electronics devices (PC, tablet, smartphone) and contained short videos that presented recognizable melodies of popular songs and a simplistic music notation staff with a disk changing its vertical position in congruence with the melody’s pitch. Musically naive participants were asked to detect occasional audiovisual mismatches between pitch and disk height, thus targeting the improvement of multisensory (audiovisual) integration. The game was developed and designed taking into account studies of the benefits of music on neuroplasticity via cognitive stimulation and emotional rewards (Koelsch, 2010), and the overall benefits (e.g., scalability, engagement, and adherence) of gamified interventions in aging (Styliadis et al., 2015; Lumsden et al., 2016). For the assessment of the training outcome, we employed a modified multi-feature oddball paradigm, in which participants were asked to attend to audiovisual incongruences while ignoring the task-irrelevant deviations of unisensory stimulus features (timbre or color; Pantev et al., 2015; Paraskevopoulos et al., 2012a, 2014, 2015). In addition, we took EEG measurements while the participants performed the odd-ball attentional task (pre and post training) to investigate the effects of the intervention on brain activity. We followed a combined ERP-spatial activation-effective connectivity approach, in an exploratory nature with no predefined time intervals or regions of interest. EEG measures are particularly relevant to study the brain mechanisms relating to attention due to their high temporal resolution (O’Brien et al., 2013; Justo-Guillén et al., 2019). To investigate spatiotemporal dynamics in relation to aging mechanisms (Grady, 2012), we further combined source reconstruction (Justen and Herbert, 2018) and functional connectivity methods (Forstmann et al., 2011; Jahfari et al., 2011; van Belle et al., 2014; Chapman et al., 2015). In a recent study, Paraskevopoulos et al. (2021) employed this methodological-analytical approach to disentangle the complex layered and interconnected system supporting multisensory training in young and older adults.

We hypothesize that multisensory integration training would strengthen top-down selective attention of the task-relevant sensory stimuli, enhancing in this way multisensory integration. This in turn would strengthen the representation of the integrated stimulus, making it more likely to win the selection competition with the task-irrelevant bottom-up information. Thus, we expected that participants’ performance in the odd-ball task (the outcome measure) will be less affected, as a result of the training, by the odd-ball uni-sensory deviant. We also expect that both groups, younger and older adults, will show enhanced performance at post-training, but given that older adults typically show enhanced multisensory integration abilities (Laurienti et al., 2006; Mozolic et al., 2012) but reduced transferability of cognitive training effects (Park and Bischof, 2013) may benefit less from the intervention.

## Materials and Methods

### Participants

Forty-four participants were recruited for this study: twenty-one young adults aged 18 to 35 years, and twenty-three older adults aged over 60 years. Inclusion criteria were: (i) a score of 26 or above the Montreal Cognitive Assessment (MOCA; Nasreddine et al., 2005), (ii) not having music training other than the compulsory music education at elementary school, (iii) the lack of a diagnosed psychiatric/neurological disorder or history of drug or alcohol abuse; (iv) being right-handed according to the Edinburgh Handedness Inventory, and (v) had normal hearing and normal or corrected-to-normal vision. Fourteen participants (6 young adults, 8 older adults) were excluded from analysis; one due to a technical problem during an EEG measurement, and the rest because they did not complete the training protocol. Thus, we report the results from thirty participants, fifteen young adults (Mean age = 22.57 ± 6.93, 7 males), and fifteen older adults (Mean age = 68.2 ± 5.46, 5 males, age range = 61–78). Participants did not receive any kind of compensation or reward for their performance. The study was approved by the Ethics Committee of Aristotle University of Thessaloniki, and all the participants provided informed written consent. The study was conducted in accordance with the declaration of Helsinki.

### Experiment

#### Stimuli

**FIGURE 1:**
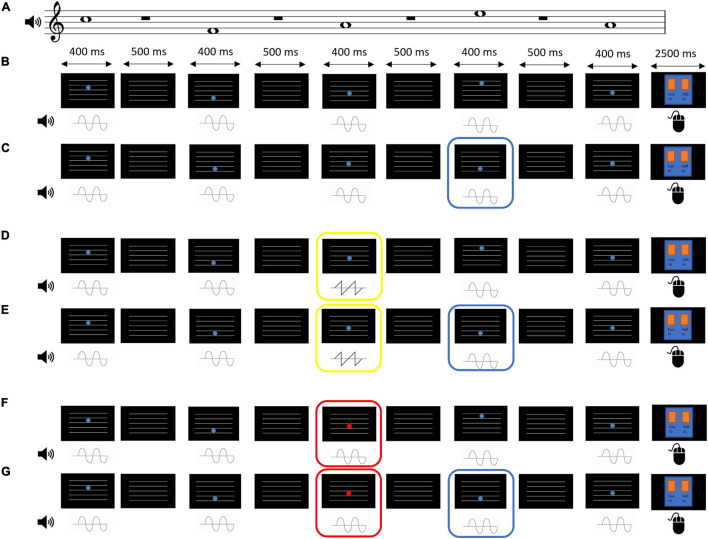
Example trials from the EEG measurement task under all the possible conditions. Each trial presented a sequence of 5 tones paired with a disk that changed its position in height. Subjects were instructed to attend to the visual (disk) and the auditory stimulus (tone) congruence according the rule “the higher the pitch, the higher the disk position”. After each sequence, they were given 2.5 s to provide an answer by using the mouse, indicating whether the sequence was “correct” (left mouse button) or “incorrect” (right mouse button). Unisensory deviants were asked to be ignored. For the purposes of illustration, all examples below have the same tone sequence. **(A)** The pitch of the tones in the examples described below. **(B)** A trial that belongs to the Audiovisual congruence type. In this type, no audiovisual incongruence and no unisensory deviance are present. **(C)** A trial that belongs to the Audiovisual incongruence type. In this type, there is no unisensory deviance but one of the disks’ height (here, the one in the 4th position of the sequence, marked by the blue frame) does not match the tone’s pitch. **(D)** A trial that belongs to the Auditory deviance type. One of the tones (here, in the 3rd position, marked by the yellow frame) has a sawtooth timbre instead of a sine. In this trial type, an audiovisual incongruence may or may not be present (50% chance), but not overlapping with the auditory deviant. This example does not contain an audiovisual incongruence. **(E)** As **(D)** above, but this example contains an audiovisual incongruence in the 4th position (marked by the blue frame). **(F)** A trial that belongs to the Visual deviance type. One of the disks (here, in the 3rd position, marked by the red frame) appears in red color instead of blue. In this trial type, an audiovisual incongruence may or may not be present (50% chance), but not overlapping with the visual deviant. This example does not contain an audiovisual incongruence. **(G)** As **(F)** above, but this example contains an audiovisual incongruence in the 4th position (marked by the blue frame).

The experimental task employed was an adaptation of an audiovisual multi-feature oddball paradigm (Näätänen et al., 2004). Instead of a long stream of stimuli, we employed distinct trials (Paraskevopoulos et al., 2014, 2021). Each trial presented a short sequence of 5 tones (Figure 1), randomly selected from a set of 4 tones that correspond to the spaces between the lines of a music notation staff (F5 = 698.46 Hz, A5 = 880.46 Hz, C6 = 1046.50 Hz, and E6 = 1318.51 Hz). To complete the 5 tones in the sequence, one of the 4 tone of the set was repeated, in a random position within the sequence but not adjacently to itself. Each tone had a duration of 400 ms, with 500 ms of silence between the tones. The whole trial lasted 2 s. The amplitude envelope of each individual tone was flat with a 10 ms fade-in and 10 ms fade-out. The audio sampling rate was 48 kHz with 16-bit resolution.

Visually, 5 horizontal white lines similar to a music staff were displayed throughout the 2 s sequence over a black screen. Each auditory tone was visually paired with a blue disk (R = 86, G = 126, B = 214) that was positioned in one of the spaces between the lines, again similar to music notation (notes F, A, C, and E). The disk was presented for 400 ms as well. During the 500 ms of silence between the tones, only the horizontal lines were visible.

The congruence between the auditory (tone sequence) and the visual (blue disk motion) stimuli, and the presence of auditory (timbre) or visual (color) deviant stimuli were manipulated. Specifically, there were four types of trials: *Audiovisual congruence*, where the position of the disks matched the tones as in standard music notation and there was no unisensory deviance (Figure 1A); *Audiovisual incongruence*, where one of the disks (chosen randomly, excluding the first and the last) did not match its accompanying tone and was positioned in a higher or lower space, that is, a drop in tone pitch was accompanied by an increase in disk position height and vice versa. Again, in this trial type there was no unisensory deviance; *Auditory deviance*, where one of the tones (chosen randomly, excluding the first and the last) was replaced by a sound at the same pitch, loudness, and duration as the standard tone, but with a sawtooth timbre with low-pass filter at 5000 Hz. Audiovisual incongruence appeared in half of the trials, but never overlapped with the auditory deviance (Figure 1B); *Visual deviance*, where one of the disks (chosen randomly, excluding the first and the last) was replaced by a red disk (R = 214, G = 86, B = 126). Again, audiovisual incongruence appeared in half of the trials, but never overlapped with the visual deviance.

Each trial was followed by a 2.5 screen that visually reminded the participants to provide a response according to the task (see below, *Procedure*).

#### Procedure

Participants were seated in a comfortable chair inside a magnetically isolated, sound-attenuated booth. Individual hearing threshold was determined with an accuracy of 5 dB at the beginning of each EEG session using the C6 stimulus tone and auditory stimuli were delivered at 60 dB SL above the defined threshold value. They undertook a two-alternative forced choice test and were asked to focus on how the pitch and disk height matched according to the rule “the higher the pitch, the higher the disk position.” After each trial, they were given 2.5 s to provide an answer of whether they spotted a violation of this rule or not by operating the computer mouse with their right hand (“correct”: left click for all-congruent trial, “false”: right click for presence of incongruence in trial). The response screen did not wait for input but was fixed at 2.5 s. No feedback was provided. Participants were instructed to ignore changes in timbre or color. There was one short practice block to familiarize the participants with the task (not included in analysis), and three experimental blocks of 128 trials each (384 trials in total). Trial type order was random, but each type was allocated with 96 trials. Participants were allowed to take a short break between blocks. The total duration of the three blocks was approximately 42 min.

#### Apparatus

The stimuli were delivered by Presentation^®^ software (Version 18.0, Neurobehavioral Systems Inc., Berkeley, CA^1^, United States) through a monitor set at 60 Hz refresh rate, positioned 110 cm away from the chair at an ± 1.15° angle vertically and ±3.86° angle horizontally and a pair of headphones (Phillips SHL3260) set at 60dB above the hearing threshold of each subject. Behavioral responses were given through the mouse by pressing either the right or left button and saved as log files on the computer.

Brain activity was recorded by a 128-active electrodes system (Nihon Kohden EEG-1200) at a sampling rate of 1000 Hz. Two 10–20 caps were used, an active (actiCAP128, Brain Products) and a passive (R-Net, Brain Products), but each participant used the same type of cap during the duration of the study. Audio-signal triggers were used to mark the onset and type of events and to synchronize the EEG recording with the presentation of stimuli.

### Intervention Protocol

#### Stimuli

The training procedure consisted of whole or recognizable parts of melodies of 42 popular Greek songs. They were arranged as single-voiced unaccompanied piano melodies with a duration of 4.92 to 8.25 s (M = 6.89, SD = 8.42) and a number of 8 to 16 notes (M = 13, SD = 2). They were made with the use of Samplitude ProX 3 software (MAGIX Software GmbH, Berlin) and its integrated virtual plugin instrument Concert Grand. All melodies were exported in mono audio with a sampling rate of 44.1 kHz, 16-bit resolution. A simplified music notation system was used for the videos, similar to the one used in the experiment. Specifically, five horizontal lines were constantly visible, and a blue disk appeared simultaneously with each note and for the same duration as the note. The disks matched the notes according to standard music notation, using up both spaces and lines on the staff. However, contrary to standard notation, each disk corresponded only to one pitch of the melody, whereas notes on a music staff can be accompanied by accidentals (#, b) indicating a different pitch. That is, notation was simplified by discarding accidentals. Melodies were carefully selected so that only diatonic ones were included, regardless of their absolute key.

Two categories of videos were made for each melody: the congruent category, where the position of the disks was correct throughout the video, and the incongruent category, where one of the disks (chosen randomly, excluding the first and the last) was displaced according to the same criteria followed in the experimental task. That is, if the melody dropped in pitch, the disk ascended in position and vice versa. The position of the incongruence, the duration of the incongruent note-disk pair as well as the duration of its previous pair were pseudo-randomized.

Four difficulty levels were created by superimposing audio and visual noise on the videos in the following manner: *Level 1*, not any kind of noise; *Level 2*, only audio white noise at −6 dB from the RMS level of the melody; *Level 3*, audio white noise at −6 dB and visual grain (“TV static”) at 75% opacity; *Level 4*, audio white noise at the same level as the RMS level of the melody and visual grain at 75% opacity. The audio in all videos was loudness matched at −16 LUFS (Loudness Unit Full Scale).

#### Procedure

Participants were free to choose their own time and place of the training sessions but were instructed to be in a quiet place without distraction. Each daily session lasted about 20 min and consisted of 55 videos (congruent or incongruent, in pseudorandom order). Participants were instructed to follow the congruence of notes and disks according to the rule “the higher the pitch, the higher the disk position” and provide an answer of whether they detected an incongruence or not by clicking on the relevant icon below the video. They received a feedback remark at each response, indicating whether their response was correct or incorrect, which was shown for 3 s before automatically proceeding to the next video. At the end of each session, a screen informed the participants of their total daily score and asked them to fill in their subject code in order for their scores to be anonymously sent to our server. There was no reward and participants could not see the anonymous scores of other participants. Each week, participants completed 5 sessions and had 2 days off. The sessions started at difficulty level 1 and automatically progressed up to level 4 week-by-week, amounting to a total of 4 weeks and 20 daily sessions. The majority of the participants (14 older adults and 13 young adults; 1 older adult and 2 young adults could not be reached) rated retrospectively the training in terms of familiarity (“*How familiar were the songs to you?*”) and likeability (“*How much did you enjoy the songs?*”) using a 9-point Likert scale (1-not at all to 10-very much).

#### Apparatus

Participants played the training game through their electronic devices (computer, smartphone, or tablet) by visiting the MusicPlast webpage^2^. The videos were uploaded to YouTube.com as “unlisted” and were embedded in the interface of the browser-based game. The webpage was made with the e-learning software Adobe Captivate (Adobe Systems Inc., San Jose, CA, United States).

### Data Analysis

#### Behavioral

To assess learning effects during the training, mean accuracy of the first two sessions the last two sessions was calculated and entered in a 2 × 2 mixed model with between-subjects factor *Group* (Younger adults, Older adults) and within-subjects factor *Session* (First two, Last two). In addition, to assess if there were differences between younger and older participants on the familiarity and likeability of the gamified training, the scores from the two self-report questions were submitted to a non-parametric Mann–Whitney test.

The outcomes measures employed from the experimental task were the participants’ Sensitivity (ability to detect audiovisual incongruence) as indexed by the *d’* (McMillan and Creelman, 2005), and the participants’ response Bias (correct vs. false) as indexed by the *c* scores (McMillan and Creelman, 2005). Specifically, three *d*’ and *c* scores for the detection of audiovisual incongruence under different conditions were produced. In the behavioral condition *Audiovisual task (AV)*, Sensitivity and Bias indices for the audiovisual task in the absence of unisensory deviants were calculated from responses in trial categories *Audiovisual congruence* and *Audiovisual incongruence*. The behavioral condition *Audiovisual task + Auditory deviance (AV+AudDev)* regarded the sensitivity and bias for the audiovisual task in the presence of auditory deviants and was calculated from responses in trial category *Auditory deviance.* Finally, the behavioral condition *Audiovisual task + Visual deviance (AV+VisDev)* regarded the Sensitivity and Bias for the audiovisual task in the presence of visual deviants and was calculated from responses in trial category *Visual deviance*. Statistical analyses were run with SPSS v25 (SPSS Inc., Chicago, IL, United States), and the normality test for the dependent variables was found satisfactory. The *d’* and c scores were submitted to two separate mixed 2 × 2 × 2 ANOVAs, with *Group* (Younger adults, Older adults) as the between-subject factor and *Time* (Pre, Post) and *Condition* (AV, AV+AudDev) or (AV, AV+VisDev) as the within-subjects factors. In order to examine potential differences in performance for visual versus auditory deviant unisensory stimuli and based on evidence suggesting temporal differences in the processing of these stimuli (see ERP analyses below), we conducted two separate ANOVAs. The auditory analysis included the *AV* and *AV+AudDev* behavioral conditions and the visual analysis included the *AV* and *AV+VisDev* behavioral conditions. Furthermore, a measure of distraction was defined in each strand as the difference of the *d’* scores between the *AV* and *AV+*deviant conditions, which was then entered in a 2 × 2 mixed model with between-subjects factor *Group* (Younger adults, Older adults) and within-subjects factor *Time* (Pre, Post), for each modality visual vs. auditory separately.

#### EEG Preprocessing

Preprocessing was performed using the software BESA Research (Version 6, Megis Software, Heidelberg, Germany). Blinks were removed using an adaptive correction method (Ille et al., 2002) and bad channels were interpolated. Further artifacts were removed through automatic artifact rejection during the averaging of trials. Epochs were defined at 1000 ms, of which 200 ms pre-stimulus and 800 ms post-stimulus. Filters applied were a high-pass filter at 2 Hz, a low-pass filter at 30 Hz and a notch filter at 50 Hz. For the purposes of the ERP analysis in a multifeature oddball paradigm, there were three conditions. In ERP condition *Standard*, the 3rd tone-disk pair of each *Audiovisual congruence* trial type was selected. ERP condition *Auditory deviant* included the timbre deviant tones that appeared in trial category *Auditory deviance*. Finally, ERP condition *Visual deviant* included the color deviant disks that appeared in trial category *Visual deviance.* Each ERP condition consisted initially of 96 trials. After applying artifact rejection, a minimum of 50 trials was ensured per condition per subject, or else the dataset was deemed unusable and was rejected for further analysis. All participants had the minimum number of trials required per condition.

#### Statistical Analysis of ERP Data

Since two electrode caps were used, averaged ERP data were transformed to the Standard-81-electrode system. Due to the exploratory and data-driven scope of our study, there were no pre-defined time intervals of interest in the ERP epoch. To search for significant differences in topographies across the epoch, ERP data, striped of the 200 ms pre-stimulus interval, were entered in 2 × 2 × 2 mixed models in the software Ragu (Koenig et al., 2011), with between-subjects factor *Group* (Younger adults, Older adults) and within-subjects factors *Time* (Pre training, Post training) and *Condition* (Standard, Auditory deviant/Visual deviant). According to our separation of the analysis in auditory and visual strand, two separate statistical analyses were run, with one containing the *Standard* and *Auditory deviant* conditions and the other the *Standard* and *Visual deviant* conditions. Topographic-ANOVA (TANOVA) tests were performed to show which time intervals exhibited statistically significant differences in scalp topographies. Data were normalized (L2 norm of the raw data) and, for the purposes of multiple comparisons corrections across epoch time points, a randomization method was used with 5000 randomizations and control of the false positive rate. Significant time intervals at corrected *p* < 0.05 were obtained for each main effect and interaction of the three factors.

#### Source Localization

Source activity was estimated by Low-resolution electromagnetic tomography (LORETA) (Pascual-Marqui et al., 1994) through the BESA software. This method was chosen based on successful prior experience of the authoring team in the same (Paraskevopoulos et al., 2021) or similar experiment paradigms (Paraskevopoulos et al., 2012b, 2014). While this previous research focused on one specific interval (130–170 ms) and found significant neuroplastic effects, the present study applied LORETA on the time intervals that resulted from the statistical analysis of the ERP data. *Standard* and *Auditory deviant* conditions source reconstructions were calculated for the time intervals that were found to be significant in the auditory strand of the ERP statistical analysis. Likewise, Standard and Visual deviant conditions source reconstructions were calculated for those time intervals found in the visual strand of the ERP statistical analysis. The head model, integrated in the BESA software, was a realistic approximation derived from 50 individual MRIs in Talairach space. Brain volume images were exported by averaging the inverse solution across time points within each time interval and then smoothed at 7 mm FWHM (Gaussian kernel).

#### Statistical Analysis of Source Images

Images were coregistered to a 2 × 2 × 2 mm MNI space using the coregistration tool in the Statistical Parametric Mapping toolbox (SPM 12^3^). The toolbox used for the statistical analysis was the Sandwich Estimator for Longitudinal and Repeated Measures Data (SwE 2.2.1) (Guillaume et al., 2014), an extension for SPM, while both run on Matlab (Version R2019a, Math Works Inc., Natick, MA, United States). Multiple separate 2 × 2 × 2 mixed model analyses were run, one for each significant time interval. The between-subjects factor was Group (Younger adults, Older adults) and the within-subjects factors were Time (Pre training, Post training) and Condition (Standard, Auditory deviant/Visual deviant). Standard and Auditory deviant conditions were tested for the time intervals found in the auditory strand of the source analysis and Standard and Visual deviant conditions were tested for those intervals found in the visual strand. The contrasts defined in this stage for each time interval, matched its respective significant effect/interaction found in the statistical analysis of the ERPs, and were tested with F-tests. The method of choice for multiple comparisons correction was a cluster-based approach with the cluster-forming threshold set at *p* = 0.1. The cluster extent threshold was calculated through the Gaussian Random Field (GRF) theory method implemented in the DPABI toolbox (Yan et al., 2016), which is similar to the GRF theory correction in FSL performed by the *easythresh* script, and is described by Friston et al. (1991, 1994). Intrinsic smoothness was estimated by DPABI from the z-transformed parametric statistic maps that SwE generated from each time interval source test. Thus, GRF-corrected cluster sizes were obtained separately for each time interval and then used for cluster-extent thresholding in its respective source test.

To explore the nature of the F-test results *post-hoc*, the eigenvalues of each cluster were extracted through the SwE toolbox and further analyzed in SPSS. Normality of data was examined with Shapiro-Wilks tests. Paired-samples *t*-tests were used for the exploration of within-subjects effects and independent samples *t*-tests for between-subjects effects.

#### Connectivity

Since the visual strand of analyses bore no significant effects on the source-space, the connectivity analysis described below regards only the auditory strand, that is, the comparison between standard and auditory deviant conditions. The statistically significant clusters found in source analysis constituted the nodes of a network. First, to obtain the time-series, LORETA solutions of the EEG data were computed for the entire post-stimulus epoch in 4-dimensional format for each subject, each time point (pre-, post-training) and each condition (Standard and Auditory deviant). Then, LORETA images were masked, and the time-series were extracted independently for each cluster. The connectivity measure that was used was Transfer Entropy, as calculated by the Matlab tool HERMES (Version 2020-04-26) (Niso et al., 2013) and an adjacency matrix of Transfer Entropy was generated for each subject, time point and condition. Transfer Entropy is a measure suitable for effective connectivity as it provides causal information. It also makes no assumptions on the dependence (whether linear or non-linear) between the time-series, making it advantageous for exploratory analyses.

Transfer Entropy matrices were statistically analyzed with the use of the Network-Based Statistic toolbox (NBS, Version 1.2) (Zalesky et al., 2010). A 2 × 2 × 2 mixed model included a between-subjects factor *Group* (Younger adults, Older adults) and the within-subjects factors *Time* (Pre training, Post training) and *Condition* (Standard, Auditory deviant). Exchange blocks were used so that permutations do not violate the longitudinal nature of the data. F-tests were run for all the main effects and interactions except for the main effect of *Group*. Due to the intrinsic nature of NBS’s model design, this effect was tested separately in a one-way ANOVA. Results are reported at *p* < 0.05 FDR-corrected with 50,000 permutations. The toolbox also offers the NBS method, a type of multiple comparisons correction similar to cluster inference but in a graph theory setting, although it provides no information as to where exactly the significant differences lie. On the other hand, the FDR, while stricter, allows inference for each individual link in the graph and is suitable for networks with a small number of nodes. Moreover, contrary to the directed nature of Transfer Entropy, the toolbox is non-directional and utilizes only the upper triangular of each participant’s adjacency matrix. Hence, tests were run twice – once normally and once on the transposed adjacency matrices. Finally, results were unified in a single connectivity matrix that contains the statistical values for each connection in a directional manner. For visualization, the toolbox BrainNet Viewer (Version 1.7^4^) was used (Xia et al., 2013). Further exploration of the results, in order to reveal the nature of the effects and interactions that were found significant, was performed directly on the participants’ Transfer Entropy scores for each connection with the use of SPSS.

## Results

### Behavioral Analysis

#### Learning and Self-Report Measures Analyses During Training

The analyses revealed a statistically significant main effect of *Session* [*F*(1, 28) = 47.35, *p* < 0.001, η_*p*_^2^ = 0.637], that is, overall accuracy was better in the last two sessions relative to the first two sessions of the training. The main effect of *Group* was also significant [*F*(1,28) = 9.64, *p* = 0.004, η_*p*_^2^ = 0.263], specifically younger adults had overall higher accuracy than older adults. The interaction between *Session* and *Group* was not significant [*F*(1, 28) = 2.093, *p* = 0.159, η_*p*_^2^ = 0.072].

The participants reported positive attitudes toward the training, both in terms of familiarity (young adults: M = 7.69, SD = 1.6; older adults: M = 8.64, SD = 1.39) and likeability (young adults: Mean = 7.23, SD = 1.87; older adults: M = 8.85, SD = 1.35). The Mann-Whitney tests revealed that the two groups differed significantly only in the likeability rating (*U* = 39.00, *p* = 0.011), with older adults finding the songs more enjoyable than younger adults, but there were no differences on familiarity rating (*U* = 59.5, *p* = 0.115).

#### Auditory Strand

**FIGURE 2:**
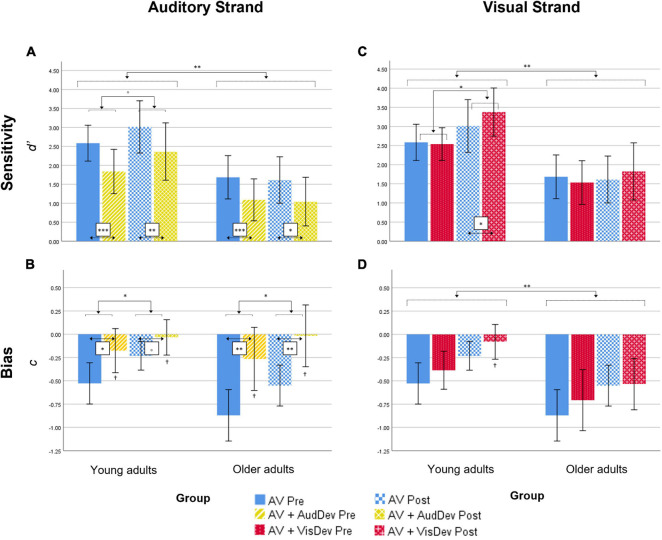
Behavioral results for both the Auditory strand (left column) and the Visual strand (right column), for both measures Sensitivity (top row) and Bias (bottom row). In detail: results for the Sensitivity **(A)** and Bias **(B)** scores in the Auditory strand of the analysis, which compares the Audiovisual task condition to the Audiovisual task + Auditory deviance condition; results for the Sensitivity **(C)** and Bias **(D)** scores in the Visual strand of the analysis, which compares the Audiovisual task condition to the Audiovisual task + Visual deviance condition. AV, score for the main audiovisual task without distractors; AV+AudDev, score for the main audiovisual task in the presence of the auditory distractor; AV+VisDev, score for the main audiovisual task in the presence of the visual distractor; Pre, before training; Post, after training. °*p* < 0.1; *p < 0.05; **p < 0.01; ***p = 0.001; ^†^non-significant bias (one-sample test against 0, *p* = 0.002). Error bars show 95% CI.

*d*’ scores analyses. Results showed a significant main effect of *Group* [*F*(1,28) = 9.03, *p* = 0.006, η_*p*_^2^ = 0.244]. Younger adults showed overall better discrimination of audiovisual congruence than Older adults (Table 1; Figure 2A), although both groups overall performed above-chance (one-sample *t*-tests per group, per condition, per time point, all *p* < 0.05). The main effect of *Condition* was also significant [*F*(1,28) = 47.57, *p* < 0.001, η_*p*_^2^ = 0.629], that is, discrimination of audiovisual congruence was worse in the deviant condition relative to the standard condition. The main effect of *Time* was not significant [*F*(1,28) = 2.25, *p* = 0.145, η_*p*_^2^ = 0.074]. The interaction *Time* by *Group* did not reach statistical significance [*F*(1,28) = 3.69, *p* = 0.065, η_*p*_^2^ = 0.116], although there was a non-significant tendency for better performance after training in the group of younger adults [*t*(14) = 1.92, *p* = 0.075]. In the group of Older adults, there were non-significant differences between the pre and post testing [*t*(14) = −0.462, *p* = 0.651]. The *Condition* by *Group* interaction and the *Time* by *Condition* by *Group* interaction were not significant [*F*(1,28) = 0.39, *p* = 0.536, η_*p*_^2^ = 0.014 and *F*(1,28) = 0.07, *p* = 0.789, η_*p*_^2^ = 0.003, respectively]

**TABLE 1:** Summary of behavioral results, for the auditory and visual strands of analysis, for both measures (Sensitivity or d’, Bias or c).

		Auditory strand			Visual strand		
Effects	Measure	*F*(1,28)	*p*	η_*p*_^2^	*F*(1,28)	*p*	η_*p*_^2^
Time	Sensitivity	2.25	0.145	0.074	6.79	**0.015**	0.195
	Bias	13.02	**0.001**	0.317	11.73	**0.002**	0.295
Group	Sensitivity	9.03	**0.006**	0.244	11.62	**0.002**	0.293
	Bias	2.55	0.121	0.084	8.24	**0.008**	0.227
Time X Group	Sensitivity	3.69	0.065	0.116	3.35	0.078	0.107
	Bias	0.21	0.646	0.008	0.12	0.736	0.004
Condition	Sensitivity	47.57	**<0.001**	0.629	1.49	0.232	0.051
	Bias	21.03	**<0.001**	0.429	6.51	**0.016**	0.189
Condition X Group	Sensitivity	0.39	0.536	0.014	0.67	0.418	0.024
	Bias	2.57	0.120	0.084	0.37	0.550	0.013
Time X Condition	Sensitivity	0.21	0.653	0.007	16.13	**<0.001**	0.366
	Bias	2.39	0.137	0.077	1.81	0.190	0.061
Time X Condition X Group	Sensitivity	0.07	0.789	0.003	0.05	0.815	0.002
	Bias	0.31	0.581	0.011	2.36	0.135	0.078

*Statistically significant results in bold.*

c scores analyses. Overall, *c* scores were negative, which reflect a general tendency in the participants for the response “correct” (Figure 2B). In order to explore whether this constituted an actual bias or not, one-sample *t*-tests were performed against 0 for each condition, each time point and each group. Bias in the *AV+AudDev* condition, both pre and post training, was not significantly different than 0, although with a high variability in the responses. The ANOVA results showed a significant main effect of *Condition*, [*F*(1,28) = 21.03, *p* < 0.001, η_*p*_^2^ = 0.429], with less negative scores (less biased responses) in the deviant condition relative to the standard condition. Interestingly, the main effect of *Time* was also significant [*F*(1,28) = 13.02, *p* = 0.001, η_*p*_^2^ = 0.317] that is, scores were also less negative (less biased responses) after the training. All other main effects and interactions were not significant [*Group*: *F*(1,28) = 2.55, *p* = 0.121, η_*p*_^2^ = 0.084; *Time* by *Group*: *F*(1,28) = 0.21, *p* = 0.646, η_*p*_^2^ = 0.008; *Condition* by *Group*: *F*(1,28) = 2.57, *p* = 0.120, η_*p*_^2^ = 0.084; *Time* by *Condition*: *F*(1,28) = 2.39, *p* = 0.137, η_*p*_^2^ = 0.077; *Time* by *Condition* by *Group*: *F*(1,28) = 0.31, *p* = 0.581, η_*p*_^2^ = 0.011)].

#### Visual Strand

*d*’ scores analyses. Results showed a significant main effect of *Group* [*F*(1,28) = 11.23, *p* = 0.002, η_*p*_^2^ = 0.293] (Figure 2C), discrimination of audiovisual congruence was overall better in Younger adults relative to Older adults. The main effect of *Time* was also significant [*F*(1,28) = 6.79, *p* = 0.015, η_*p*_^2^ = 0.195], indicating an improvement in performance after training. The interaction *Time* by *Condition* was also significant [*F*(1,28) = 16.13, *p* < 0.001, η_*p*_^2^ = 0.366]. To analyze the interaction, we collapsed the data across Group, and conduced planned *t*-tests for each time condition. At pre-training, there were no differences between the standard and deviant conditions [*t*(29) = 1.024, *p* = 0.314], while at post-training, sensitivity in the presence of the visual deviant stimulus was greater than in the undistracted audiovisual task [*t*(29) = 3.36, *p* = 0.002]. The interaction *Time* by *Group* did not reach statistical significance [*F*(1,28) = 3.35, *p* = 0.078, η_*p*_^2^ = 0.107]. However, planned *t*-tests, on collapsed scores across condition, showed significantly better performance after training only in the group of younger adults [*t*(14) = 2.58, *p* = 0.022, and *t*(14) = 0.762, *p* = 0.458, for Older adults]. The main effect of *Condition*, the *Condition* by *Group* interaction and the *Time* by *Condition* by *Group* interaction were not significant [*F*(1,28) = 1.49, *p* = 0.232, η_*p*_^2^ = 0.051; *F*(1,28) = 0.67, *p* = 0.418, η_*p*_^2^ = 0.024; *F*(1,28) = 0.05, *p* = 0.815, η_*p*_^2^ = 0.002, respectively].

c scores analyses. Results showed statistical significance in all main effects: [*Group: F*(1,28) = 8.24, *p* = 0.008, η_*p*_^2^ = 0.227; *Time: F*(1,28) = 11.73, *p* = 0.002, η_*p*_^2^ = 0.295; *Condition*: *F*(1,28) = 6.51, *p* = 0.016, η_*p*_^2^ = 0.189] (Figure 2D). That is, scores were overall less negative in the group of younger adults, after the training, and in the presence of the visual deviant. None of the interactions reached statistical significance [*Time* by *Group*: *F*(1,28) = 0.12, *p* = 0.736, η_*p*_^2^ = 0.004; *Condition* by *Group*: *F*(1,28) = 0.37, *p* = 0.550, η_*p*_^2^ = 0.013; *Time* by *Condition*: *F*(1,28) = 1.81, *p* = 0.190, η_*p*_^2^ = 0.061; *Time* by *Condition* by *Group*: *F*(1,28) = 2.36, *p* = 0.135, η_*p*_^2^ = 0.078]. In general, responses were biased toward the “correct” response, but less so for younger adults, in post-training under the deviant condition (one-sample *t*-test against 0, *p* = 0.37; all other *p* < 0.05).

#### Distraction

**FIGURE 3:**
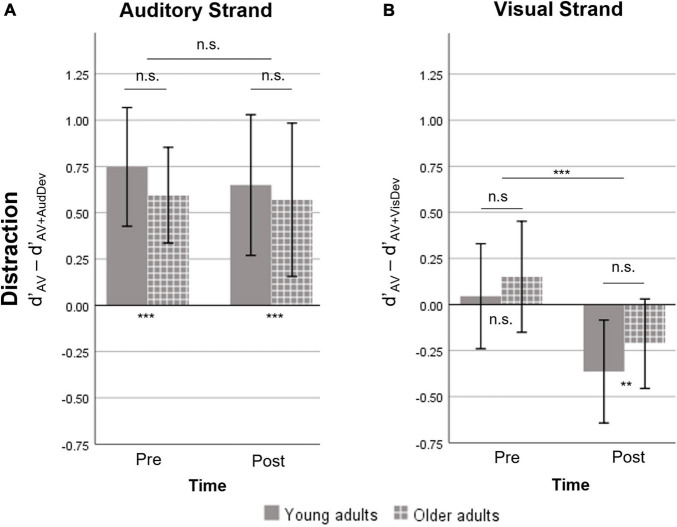
Measure of distraction, defined as the difference in d’ between the AV and one deviant condition. A positive value indicates the costs on Sensitivity induced by the presence of a deviant stimulus. A negative value indicates a facilitation effect induced by the deviant stimulus. **(A)** The auditory strand containing the AV and AV+AudDev conditions. At both timepoints, the auditory deviant distracted participants of both groups. **(B)** The visual strand, containing the AV and AV+VisDev conditions. The visual deviant stimulus was non-distractive pre-training and facilitating post-training, for both groups. AV, score for the main audiovisual task without distractors; AV+AudDev, score for the main audiovisual task in the presence of the auditory distractor; AV+VisDev, score for the main audiovisual task in the presence of the visual distractor ^∗∗^*p* < 0.01; ^∗∗∗^*p* ≤ 0.001; n.s. = non-significant comparison. Error bars show 95% CI.

The repeated measures ANOVA of the distraction measure showed no significant effects or interactions, except for a *Time* effect in the visual strand (Figure 3). Specifically, there was significant distraction caused by the presence of the auditory deviant, which was not modulated by training (*p* > 0.5) or age (*p* > 0.5). On the other hand, there was an effect of training on distraction due to the visual deviant [*t*(29) = −4.08, *p* < 0.001]. However, the visual deviant induced virtually no distraction at pre-training [*t*(29) = 1.02, *p* = 0.314], and facilitated performance instead at post-training [*t*(29) = −3.36, *p* = 0.002]. No significant *Group* effect or *Group* X *Time* interaction was found.

### Source Analysis

**FIGURE 4:**
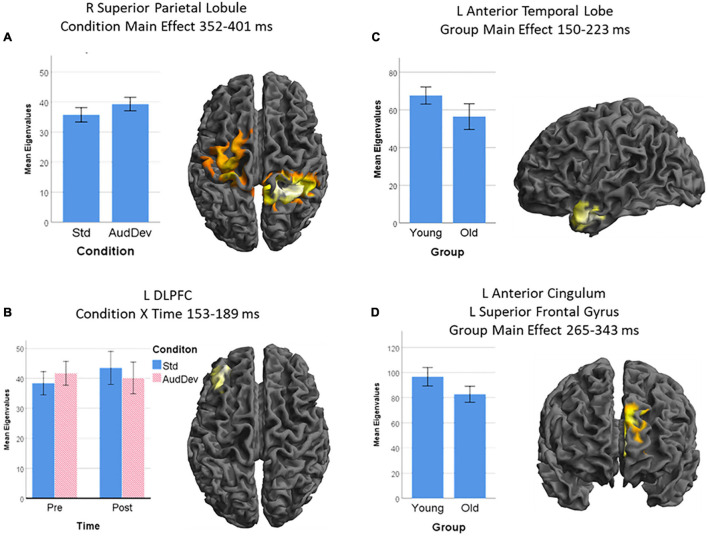
Source-level results of the statistical analysis of the LORETA images. Only the Auditory strand of analysis yielded source-level results, with four clusters: **(A)** a cluster over the right superior parietal lobule and the left pre- and post-central gyrus, being more activated in the Auditory deviant condition than the Standard condition; **(B)** a cluster over the left dorsolateral prefrontal cortex (DLPFC), being more activated after training but only in the Standard condition (condition × time interaction); **(C)** a cluster over the anterior part of the left temporal lobe, being more active in younger adults than older adults; and **(D)** a cluster over the left superior frontal gyrus extending medially to the left anterior cingulum, being more activated in younger adults than older adults. Cluster-extent thresholds corrected by GRF; cluster-forming threshold *p* = 0.01. Graphs show *post hoc* analysis of extracted eigenvalues. Error bars show 95% CI.

The statistical analysis revealed four clusters with sizes above the cluster-extent thresholds obtained from the GRF theory method. All four clusters belonged to the auditory strand of analysis, which compares the auditory deviant to the standard condition in the *Group* by *Time* by *Condition* model. Some clusters appeared in the visual strand of analysis, but none survived the GRF correction. The following report regards only the auditory strand. A summary of the results can be found on [Table 2] and visualized on (Figure 4).

**TABLE 2:** Summary of results from the statistical analysis of the source images.

Effect	Area	Peak voxel coordinates (MNI)			Peak voxel statistic	Cluster size (voxels)
		x	y	z	*X*	
Condition	R Parietal + L Pre/Post-Central Gyrus	30	−46	66	12.51	8351
Time X Condition	L DLPFC	−36	50	30	8.13	1391
Group	L Anterior Temporal Lobe	−46	−4	−36	8.70	1638
Group	L SFG + ACC	−8	46	16	11.15	2358

*Cluster-based inference; cluster-forming threshold *p* = 0.01; GRF-corrected cluster-extent thresholds; voxel size = 2 × 2 × 2 mm. DLPFC, dorsolateral prefrontal cortex; SFG, superior frontal gyrus; ACC, anterior cingulate cortex.*

The first significant cluster, which derives from the main effect of *Condition* during the time interval 352–401 ms, is located in an extended area over the right superior parietal lobule and the left precentral and postcentral gyri with the peak voxel being in the parietal portion (peak voxel x = 30, y = −46, z = 66, *X* = 11.10, cluster size = 8351). The second cluster derives from the interaction of *Time* X *Condition* in the time interval of 153–189 ms and is located in the left middle frontal gyrus, particularly the area known as dorsolateral prefrontal cortex (DLPFC) (peak voxel x = −36, y = 50, z = 30, *X* = 8.13, cluster size = 1391). The main effect of Group produced two clusters. One, stemming from the time interval 150–223 ms, is located in the left anterior temporal lobe, including the temporal pole and the anterior parts of the superior, middle, and inferior temporal gyri (peak voxel x = −46, y = −4, z = −36, *X* = 8.70, cluster size = 1638). Lastly, the other cluster from the main effect of *Group* during the 265–343 ms, is located in left frontal areas, particularly the superior frontal gyrus extending inward to the anterior cingulate cortex (peak voxel x = −8, y = 46, z = 16, *X* = 11.15, cluster size = 2358).

*Post-hoc*, the analysis of the eigenvalues extracted from each cluster showed the following: Cluster 1 (R SPL) is more activated in the *Auditory Deviant* condition than the *Standard* [*t*(29) = 4.03, *p* < 0.001]. The interaction *Time* X *Condition* in which cluster 2 (L DLPFC) was involved had to be broken down. A *t*-test of the interaction showed that the difference due to training (Post – Pre) under the *Standard* condition was greater than those under the *Auditory deviant* condition [*t*(29) = 2.82, *p* = 0.009]. Testing each condition separately showed that only the increase in the *Standard* condition came close to statistical significance [*t*(29) = 1.93, *p* = 0.063] while the decrease in the *Auditory deviant* condition was clearly insignificant (*p* = 0.58). In both clusters 3 (L AntTempL) and 4 (L SFG+ACC), Younger adults showed greater activity than Older adults [*t*(28) = 2.92, *p* = 0.007, and *t*(28) = 3.07, *p* = 0.005, respectively].

### Connectivity

**FIGURE 5:**
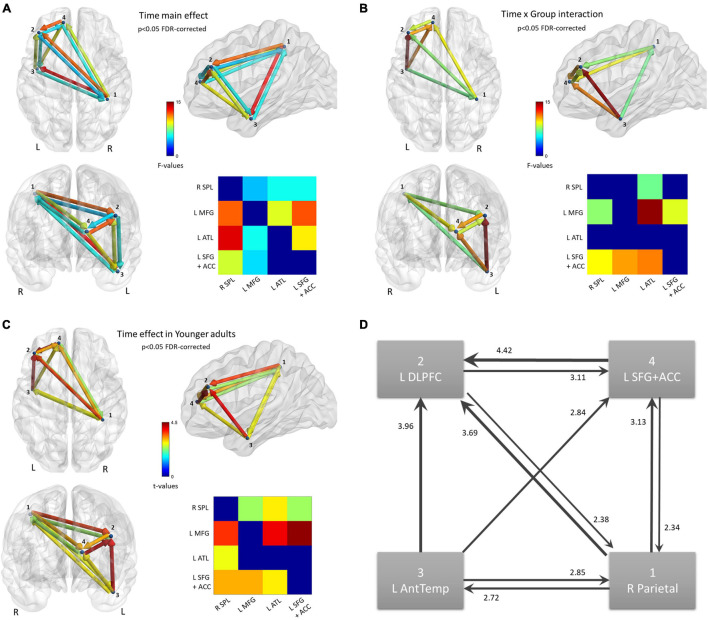
Functional connectivity results. Nodes of the network were defined based on the findings of the source activation analysis in the Auditory strand of analysis. Flow of information is measured by Transfer Entropy. **(A)** Network that increases Transfer Entropy after training, for all subjects; *p* < 0.05 FDR-corrected. **(B)** Network connections that show a statistically significant Time × Group interaction; *p* < 0.05 FDR-corrected. **(C)** Time effect on the network, revealed from the new model that includes only the Young group; *p* < 0.05 FDR-corrected. **(D)** Schematic of the Time effect in **(C)**. Edges are weighted by *t*-values, also shown as numbers.

Continuing from the source analysis, where no statistically significant clusters were found in the visual strand, only the auditory strand is relevant in the following connectivity analysis. The 4 nodes discovered in the auditory strand of the source analysis were defined as the nodes of a network. Statistical analysis of the 4 × 4 matrices of the Transfer Entropy measure revealed a main effect of *Time* (*p* < 0.05 FDR-corrected) (Figure 5A), while an also significant *Time* by *Group* interaction (*p* < 0.05 FDR-corrected) demanded further exploration of the nature of the training effects (Figure 5B). No other significant effects or interactions were found.

**FIGURE 6:**
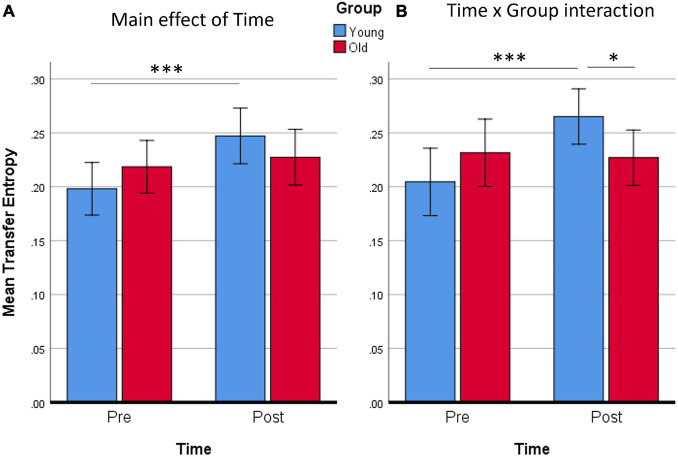
Averaged Transfer Entropy of all supra-threshold connections of the network for the main effect of Time **(A)**, or for the Time × Group interaction **(B)**. It is evident from both contrasts that only the Young group increased Transfer Entropy. ^∗^*p* < 0.05; ^∗∗∗^*p* ≤ 0.001. Error bars show 95% CI.

The mean Transfer Entropy scores were then calculated from all the significant connections included in the *Time* main effect. Subsequent *t*-tests revealed that the training affected Younger adults [*t*(14) = 4.20, *p* < 0.001] but not Older adults (Figure 6). No group differences were found either pre- or post-training. When Transfer Entropy scores were calculated from the significant connections included in the *Time* by *Group* interaction, training again seemed to affect only Younger adults [*t*(14) = 4.39, *p* < 0.001] and not Older adults. Group differences were found only post-training [*t*(28) = 2.15, *p* = 0.040]. Thus, the connectivity analyses support that there was an effect of training (*Time*) only in the Younger adult group, which is in agreement with the non-significant tendencies found in the behavioral data analyses.

In order to visualize the changes due to training in Younger adults’ connectivity, a new model was set up in the NBS toolbox. This model contained only the Younger adults, with their within-subjects data averaged over *Condition*. A *t*-test (Post > Pre) was run, again thresholded at FDR-corrected *p* < 0.05 (Figure 5C). The changes in the network of the Younger adults can be summarized as an increase in Transfer Entropy in all connections of the network except the connection directed from the two frontal nodes to the temporal node (Figure 5D). In other words, the flow of information is strengthened in a loop comprised of the parietal and frontal areas while the temporal node increases the sending of information to all other nodes and the reception of information from only the parietal node.

## Discussion

In this study, we examined how a training protocol that targets multisensory integration affects attentional capture by unisensory deviants within a multisensory task. Using a cross-sectional design, we also explored age-related differences in the training outcome. The training protocol consisted of a computerized music-reading serious game with material from popular songs lasting 4 weeks. Participants were also assessed (behavioral and EEG measures) with a multifeature oddball paradigm at pre and post training, where they had to detect audiovisual incongruences while ignoring interspersed infrequent changes in unisensory stimulus features (timbre or color). The analysis of brain data, collected by high-density EEG, followed an exploratory step-by-step approach. First, we assessed differences in scalp topographies of ERPs, then localized and analyzed the source activity for each significant time interval and finally analyzed the directed functional connectivity between those areas.

### Behavioral Results

Overall, participants showed learning effects during the training, but transfer effects to the experimental task (sensitivity measure) were restricted to younger adults, and specific conditions; that is, the visual deviant facilitated performance at post-training. This surprising facilitation and the differentiation of auditory and visual deviants will be discussed separately below. However, training did produce significant transfer effects on the response bias measure, in both the visual and auditory analyses, which were independent of age group. That is, responses were overall less “liberal” at post-training. The significant change to a more “cautious” response criterion after the training seems to be attributable to multisensory integration enhancements, by increasing the top-down readiness for dealing with a predicted incongruence. It could also be that the statistical probabilities (50% congruent 50% incongruent) and the presence of feedback during the training induced a more balanced response strategy at post-training.

Regardless of training, older adults overall showed worse performance (lower sensitivity) than younger adults in the oddball task (outcome measure). That is, they were less able to detect audiovisual incongruences. This finding is in contrast with previous studies that support the contention of enhanced multisensory integration in older age (Mozolic et al., 2012). It could possibly be explained by the speed-accuracy tradeoff, according to which older adults take longer to respond in order to compensate for their poorer accuracy (Forstmann et al., 2011; Kopp et al., 2014; Kardos et al., 2020). This hypothesis could not be tested in our study due to its EEG design. Older adults also appeared to be more biased which suggests that they adopted a more “liberal” strategy (McMillan and Creelman, 2005). They showed a stronger tendency to the response “correct” than Younger adults. This finding may be related to the finding of reduced sensitivity. That is, it could be that a reduced sensitivity to perceive multisensory incongruences may have resulted in a more “liberal” response bias.

However, we did not find age-related differences in deviance distraction. Although there is evidence suggesting age-related declines in top-down attentional processes (Gazzaley et al., 2005), our findings are in agreement with previous studies that have employed similar paradigms and have reported no age-related differences in distraction by novel deviant stimuli which do not bear cognitive/semantic interference (Alain and Woods, 1999; Beaman, 2005; Bell and Buchner, 2007; Andrés et al., 2008; Guerreiro et al., 2010; Parmentier, 2014; Leiva et al., 2015).

### Source Level

On the source level, the visual strand yielded no results from the analysis of the significant ERP intervals, possibly because of the different sensitivities of each step. In particular, there were a few clusters in the visual strand that did not pass the multiple comparisons correction, although the ERP TANOVA found significant time intervals for all the main effects. It is possible that the overall approach was not sensitive enough to capture all the dynamics in the visual strand, and more focused studies should be carried out, with hypothesis-driven time intervals or regions of interest. In the auditory strand, there are two brain areas involved in overall group differences, with greater activation in younger adults than older adults. One is the left anterior temporal lobe which is known for its multisensory functions, especially binding auditory and visual information during speech processing (Visser et al., 2012; Fan et al., 2014; Xu et al., 2015; Vigliocco et al., 2020). The other is a left frontal area, comprising of the left superior frontal gyrus which is associated with working memory performance (Alagapan et al., 2019), and the anterior cingulate cortex which is implicated in numerous aspects of cognitive control and conflict monitoring (Irlbacher et al., 2014; Widge et al., 2019). While many studies find an overactivation of frontal areas in older adults, results are conflicting (Grady, 2012). The present study falls in line with studies that find deactivations in frontal areas (Davis et al., 2008). A superior parietal area was activated in the auditory deviant compared to standard stimuli, during the 352–401 ms interval, which fits the characteristics of a P3 generator (Polich, 2007), indicating a capture of attention. Interestingly, the effects of our training protocol in spatial activity were undifferentiated between the two groups. Activity in the left dorsolateral prefrontal cortex (DLPFC) was enhanced post-training compared to pre-training, for both groups. This area is known not only for its role in cognitive control (Widge et al., 2019) and working memory (Evangelista et al., 2020), but is also thought to modulate the auditory MMN (Wang et al., 2020) as well as other ERP components (Dubreuil-Vall et al., 2019). This effect was evident only for the *Standard* condition and not the deviant, possibly indicating modulatory top-down mechanisms that strengthened after training.

### Connectivity

Our functional connectivity analysis revealed change due to training in the directed connectivity of the network, although only in the Younger adults. Generally, the result can be described as a strengthening of the interconnection between fronto-parietal areas, a strengthening of the interconnection between the temporal and the parietal area, and a strengthening of forward connections from the temporal to frontal areas. Subparts of our defined network are already outlined in existing research. The involvement of fronto-parietal networks in attentional top-down and bottom-up processes (Corbetta and Shulman, 2002) as well as in the proactive and reactive modes of control (van Belle et al., 2014) are well established. The communication between dACC and DLPFC appears to be crucial in conflict resolution and cognitive control (Irlbacher et al., 2014; Widge et al., 2019). Also, a medial-to-lateral prefrontal communication has been found to mediate the processing of response feedback (Smith et al., 2015), relevant to our training protocol which provided subjects with feedback on their responses. Known generators of the auditory MMN in frontal and temporal areas have been found to be functionally connected (Choi et al., 2013). Overall, these results support an interplay between bottom-up and top-down attentional processes, with the multisensory (temporal) area updating all the others in a bottom-up fashion, while it is being top-down regulated by the fronto-parietal network via the parietal lobe.

### DLPFC and Top-Down Control

Increased activity of the left DLPFC as a result of training was found only under the standard condition. One possible interpretation, in light of the dual mode of control (DMC) framework (Braver, 2012), is that this area promotes the proactive mode of control and the active engagement with the task (detection of audiovisual incongruence) while the unisensory task-irrelevant auditory deviant forces a shift to a reactive mode. Indeed, in a recent rTMS study, the left DLPFC was found to be associated with the proactive mode of control (Pulopulos et al., 2020). The area is thought to hold representations of active goal information and is part of a network involved in the proactive mode, along with pre-SMA and parietal areas (Irlbacher et al., 2014). However, DLPFC activity in our study may be negatively associated with the proactive mode. Based on our behavioral findings, subjects were largely biased toward the “correct” answer, which may be considered as a form of proactive control, suppressing disturbances caused by the incongruences. However, this performance is actually sub-optimal, and a training effect may have shifted this liberal strategy to a better, more neutral one. Thus, increased activity in DLPFC may be associated with less proactive control, although in a meaningful manner. The link of DLPFC to the proactive mode of control in our study can also be corroborated by the early, pre-attentive time interval (153–189 ms) during which this area was localized. It remains worth exploring the idea that this area may ameliorate -if necessary- an overly biased anticipatory mechanism. The DMC framework often regards the proactive and reactive modes as a continuum with several brain areas overlapping in both modes (the DLPFC being one of them), while promising results regarding their differentiation rest in their temporal dynamics and the spatial subdivision of each area into smaller parts (Irlbacher et al., 2014; van Belle et al., 2014). Thus, a possible task-specific and time-locked modulatory nature of the DLPFC along this continuum remains a hypothesis worth exploring.

### Multisensory vs. Unisensory Domains

From the scope of multisensory research, it is worth noting that our findings comprise of multisensory and higher cognitive areas but not unisensory areas. There is an ongoing debate on whether perceptual learning in the multisensory domain can be transferred to the constituent unisensory domains and vice versa, and where in the brain these transfers may be located. Our study is in line with the framework proposed by Proulx et al. (2014), according to which the high vs. low areas distinction is dependent on two factors, task complexity and stage of training. Easier tasks, even on an untrained stage, need only make use of the pre-existing representations in higher areas. On the other hand, more difficult tasks demand the recruitment of unisensory processing areas alongside the higher ones. We argue that our task utilized easily detectable unisensory deviants, salient enough to diminish the need for dedicated unisensory processing. Thus, we claim that the training protocol, which aimed at multisensory integration, did show certain generalizability toward the processing of unisensory information, although limited to higher order and multisensory areas and to the Younger adult group. Importantly, this only regards the processing of the auditory deviant, as the visual strand of analysis yielded no source level results and thus no network could be defined.

The generalizability partly reflects to the behavioral results, but it is important to remark that our design does not allow for a direct comparison of behavioral and EEG analyses. Our behavioral measures under the deviant conditions regard the execution of the main task in the presence of task-irrelevant stimuli and not the explicit processing of those unisensory deviants. Thus, the question of whether training in multisensory integration can generalize to unisensory processing *per se*, while central to multisensory research, cannot be addressed by our behavioral data. However, our findings do have implications for the question of whether training in multisensory integration can mitigate distraction effects caused by unisensory deviants or generalize to more cognitive domains. We conclude that our training protocol was effective in improving higher-level areas and functions, but only in the auditory strand (see below).

### Auditory – Visual Deviant Differential Effects

The auditory and visual conditions share largely the same training effects on the response bias measure, but they showed different effects on the sensitivity measure. At baseline, the visual deviant does not interference with performance, which is in agreement with previous studies that reported less, or even no distraction, from visual deviants relative to other modalities, such as auditory, tactile or multisensory distractors (Boll and Berti, 2009; Broadbent et al., 2018; Lee et al., 2018; Lunn et al., 2019; Rau et al., 2020). Studies also support that this modality differentiation is not affected by age (Downing et al., 2015), and that under certain conditions, a visual deviant can facilitate performance in an auditory task (Boll and Berti, 2009; Lee et al., 2018). We found that the visual deviant facilitated discrimination of the multisensory incongruence but only after training, and regardless of age group. One possible explanation for the different effects of the auditory and visual stimuli is the modality dominance. In a paradigm similar to our main task but without the unisensory deviants, Paraskevopoulos et al. (2015) found that there was a visual dominance in the integration of audiovisual information, as evidenced by a network along a dorsal pathway with significant contributions from occipitoparietal areas. They also found that this network was reorganized by the long-term extensive training of musicians, resulting in the involvement of more temporal areas and auditory dominance in multisensory processing. Thus, it is possible that, for non-musicians, the visual deviants draw attention and allocate more resources to the dominant modality network, which is already active in engaging with a multisensory task, conferring this way no overall performance costs. This is further supported by the visual dominance found in the study by Downing et al. (2015), and the finding that musicians are less easily distracted than non-musicians in auditory tasks with auditory distractors (Berti et al., 2006; Bialystok and DePape, 2009; Kaganovich et al., 2013). That task-irrelevant information in the dominant modality may be less costly remains to be further studied. One has to note, also, that the addition of a control group in the present work would elucidate causality issues in the training effects within each group, although the interpretation of the results that describe the comparison between young and older adults is not affected by the lack of a control group.

### Common Activation Strength Increases but Flow of Information Increase Only in Young Adults

An important finding is the differentiated training outcomes between age groups at the neural level. Specifically, older adults showed a limited capacity for improvement. It is known that older adults show reduced functional connectivity in attentional and working memory tasks and that contrasting findings have been found regarding the compensatory direction of spatial activity of the prefrontal cortex (Grady, 2012). Here we found no direct group differences in functional connectivity, but there was an improvement of functional connectivity due to training only in Younger adults. Activity of a dorsomedial frontal area was found to be generally decreased in Older adults, although both groups increased activity in a dorsolateral frontal area after training. Taken together, these results indicate that older adults may increase activity strength in individual brain areas although they show a limited capacity to increase information flow between those areas. This finding agrees with a previous study which used the same paradigm and compared young and older adults’ performance on the same task (Paraskevopoulos et al., 2021). Nevertheless, the overall finding of increased neural activity in the DLPFC is especially encouraging for the group of older adults, since it has been suggested that the structural integrity of the DLPFC is a predictor of healthy aging in the n-back task (Evangelista et al., 2020).

### Conclusion

We aimed to explore the interaction between attention and multisensory integration, which is a topic relatively understudied. Specifically, we aimed at investigating how this interaction would be modulated by multisensory training and older age. Results showed no age-related differences in behavioral distraction by visual or auditory deviants. The effect of training was modulated by the modality of the deviant stimuli. While there were no effects with auditory distraction, visual deviant stimuli facilitated performance at post-training. These differences were explained in terms of modality dominance. Multisensory training also had a significant effect on response criterion, which resulted in more cautious responses at post-training. At the neural level, neuroplastic changes regarding the auditory deviant were evident in both groups, although partly differentiated. Older adults’ changes were limited to activity strength in the left DLPFC whereas younger adults increased both activity strength in the left DLPFC and flow of information in a network comprising of a fronto-parietal subnetwork and a temporal multisensory area. The finding of higher-level areas but not auditory processing areas is in line with multisensory research and the reverse hierarchy model, under the term of less task complexity. We conclude that multisensory training protocol improved resistance to interference from unisensory deviants by reducing the response bias and possibly enhancing top-down control.

## Data Availability Statement

The datasets presented in this study can be found in online repositories. The names of the repository/repositories and accession number(s) can be found below: git@gin.g-node.org:/parasvag/MusicPlast.git.

## Ethics Statement

The studies involving human participants were reviewed and approved by Ethics Committee of the Aristotle University of Thessaloniki. The patients/participants provided their written informed consent to participate in this study.

## Author Contributions

AK authored the manuscript, designed the study, collected, analyzed, and interpreted the data. NC designed the study and collected the data. MK collected the data. GP designed the study. AV critically revised the manuscript. PB designed the study and interpreted the data. EP conceived and designed the study, collected, analyzed and interpreted the data, and critically revised the manuscript. All authors contributed to the article and approved the submitted version.

## Conflict of Interest

The authors declare that the research was conducted in the absence of any commercial or financial relationships that could be construed as a potential conflict of interest.

## Publisher’s Note

All claims expressed in this article are solely those of the authors and do not necessarily represent those of their affiliated organizations, or those of the publisher, the editors and the reviewers. Any product that may be evaluated in this article, or claim that may be made by its manufacturer, is not guaranteed or endorsed by the publisher.

## Footnotes

1
www.neurobs.com

2
http://parasvag.webpages.auth.gr/MUSICPLAST_new/

3
https://www.fil.ion.ucl.ac.uk/spm

4
http://www.nitrc.org/projects/bnv/
